# Vicarious trauma, coping strategies and nurses' health outcomes: An exploratory study

**DOI:** 10.3934/publichealth.2024055

**Published:** 2024-10-17

**Authors:** Ilenia Piras, Vanessa Usai, Paolo Contu, Maura Galletta

**Affiliations:** 1 Clinical Trials Sector, Medical Directorate, Microcythemia Hospital “A. Cao”, ASL Cagliari, Italy; 2 Intensive Care Unit, Santissima Trinità Hospital, ASL Cagliari, Italy; 3 Department of Medical Sciences and Public Health, University of Cagliari, SS554 Bivio Per Sestu, 09042 Monserrato, Cagliari, Italy

**Keywords:** vicarious trauma, impact event, mental disorders, cross-sectional study, COVID-19, moderating role, coping strategies

## Abstract

**Background:**

The COVID-19 outbreak played a significant psychological impact on nurses, as they coped with intense emotional and cognitive demands, in a context in which the Health System was not prepared to face the emergency. Literature showed that pandemics influenced the nurses' stress and psychosocial health due to poor rest, high work overloads, a lack of control over the patient flows, and a frequent isolation from family. Under these circumstances, nurses experienced severe psychological and mental stressors that generated mental health problems. Recent literature showed that coping strategies, especially those that were positive, promoted mental health in workers and helped them to face stressors.

**Objective:**

The study aimed to investigate the relationship between vicarious traumas and the impact of traumatic events on nurses' mental health. In addition, we analyzed the role of coping strategies in moderating the effect of vicarious traumas on mental health.

**Methods:**

The study was performed in November 2020, during the first wave of the COVID-19 pandemic. A self-reported structured questionnaire was administered via an online method to reduce face-to-face contact. Logistic regressions were conducted to analyze the relationship between both vicarious traumas and the impact of traumatic events impact and mental health. An interaction analysis with the PROCESS macro was performed to analyze the role of coping strategies in moderating the relationship between vicarious traumas and mental health.

**Results:**

A total of 183 nurses answered to the questionnaire. A moderation analysis showed that positive coping strategies such as physical activity, reading/music, and yoga/meditation showed to be protective in reducing the effect of vicarious traumas on the nurses' mental health problems. Conversely, negative coping strategies strengthened that relationship and may compromise their quality of working life.

**Conclusion:**

These findings provide further support for considering positive coping strategies as an important resource to alleviate psychological distress, thus helping the professional to reduce the negative effects of stress.

## 1. Introduction

A vicarious trauma is a psychological phenomenon of great concern to health care professionals, especially nurses [1], due to their close and prolonged interactions with suffering individuals. In fact, vicarious traumas or secondary traumatic stress [2] refer to the emotional and psychological impact of caring for patients who have experienced trauma. As a result, nurses are at a high risk for developing vicarious traumas due to their emotionally demanding job, which requires an intense emotional involvement and emotional labor [3]. This phenomenon can lead to profound psychological changes that manifest as physical and emotional symptoms. These include distress, anxiety, depression, and even burnout [4],[5], which can ultimately affect a nurses' well-being and ability to provide quality care [6].

Over the past three years, the COVID-19 pandemic has had a significant psychological impact on nurses as they coped with intense emotional and cognitive demands. Literature has shown that pandemics have affected a nurses' stress and psychosocial health due to poor rest, work overload, a lack of control over the patient flow, and a frequent isolation from family [7]. Under these circumstances, nurses were at risk of developing vicarious traumas [8].

The coping strategies of nurses have been identified as crucial factors in mitigating the psychological and emotional effects of these traumatic experiences [9]. Emerging evidence suggests that coping mechanisms play a pivotal role in buffering the negative effects of traumatic events, such as fear, during pandemics on a nurses' mental health [10]. Effective coping strategies are essential to manage their individual stress levels [11]. These strategies can be categorized as either positive or negative. Under stressful conditions, individuals who use positive coping strategies engage in constructive thinking and problem solving [12]. In contrast, negative coping strategies, also known as palliative coping, involve negative appraisals and avoidance behaviors. The literature suggests that nurses who use positive coping strategies experience better job performance and greater job satisfaction, which also contributes to patient safety [13],[14]. Recent literature showed that physical activity is one of the coping strategies that can promote mental health in workers and help them to face stressors [15]. Therefore, the use of positive coping strategies during the COVID-19 pandemic may improve a nurses' mental health.

In Italy, studies that addressed the issue during the pandemic were poor and mainly focused on detecting the presence of vicarious traumas [16]. This study aims to investigate the relationship between vicarious traumas and the impact of traumatic events on a nurses' mental health. In addition, we analyze the role of coping strategies in moderating the relationship between vicarious traumas and mental health problems.

## 2. Materials and methods

### 2.1. Participants and data collection

The study involved nurses who worked in a region of Central Italy, in either a hospital or a community setting, during COVID-19 pandemic. The study was carried out in November 2020, during the first wave of the pandemic. To reduce face-to-face contact, the nurses were recruited via an online survey through social channels of nursing groups from Southern Sardinia. The study aim was explained to the group administrators who authorized the survey. The nurses voluntarily and anonymously participated in the study. To collect data, a structured questionnaire was administered through a Google form online platform. Informed consent was requested from the participants before they completed the questionnaire.

### 2.2. Instrument

The questionnaire included the following validated scales from the literature.

Impact event scale-Revised (IES-SCALE-R) by Weiss and Marmar [17]: this scale evaluates an individual's stress caused by traumatic events. It includes 22 items with three sub-dimensions: avoidance (8 items), intrusiveness (8 items), and hyperarousal (6 items). The Likert scale ranged from 0 (not at all) to 4 (extremely). Sample items were “I was aware that I still had a lot of feelings about it, but I didn't deal with them” for avoidance, “I found myself acting or feeling like I was back at that time” for Intrusiveness, and “I had trouble concentrating” for hyperarousal.

Vicarious Trauma Scale by Vrklevski and Franklin [18]: this was used to analyze the level of vicarious traumatization. It includes 8 items. The Likert scale ranged from 1 (strongly disagree) to 7 (strongly agree). A sample item was “My job involves exposure to distressing material and experiences”.

Coping strategies: we used an adapted version of the instrument by Vrklevski and Franklin [18]. A total of five methods were listed to know which coping strategies the participants used to deal with job stress. We divided these strategies into positive and negative coping strategies. Three indicators were used for positive coping (physical activity, reading/music, and meditation/yoga) and two indicators were used for negative/avoidance coping (medication use and alcohol/drug use). The Likert scale ranged from 1 (never) to 4 (always). A sample item for positive coping was “Do you regularly perform physical activity (sport, exercice, yoga...)?”.

Symptom Checklist-90-Revised by Derogatis and Savitz [19]: we used three dimensions from the questionnaire to analyze main referred symptoms or problems, which included symptoms such as somatization (12 items), anxiety (10 items), and sleep disorders (3 items). The Likert scale ranged from 0 (*not at all*) to 4 (*very much*). Sample items included the following: “Headaches, Faintness or dizziness, Pains in heart or chest...” for somatization, “Suddenly scared for no reason, Feeling fearful...” for anxiety, and “Trouble falling asleep, Awakening in the early morning...” for sleep disorders.

### 2.3. Ethical statement

Because this study was observational and did not involve drugs, a formal ethical approval was not required. Italian Law 211/2003 and GDPR 2016/679 exempted non-interventional studies from the definition of medical/clinical research, which usually requires approval. Therefore, approval by a Medical Ethics Review Committee was not necessary for this study.

### 2.4. Data analysis

Data were analyzed using the SPSS software (SPSS Inc., Chicago, IL, USA), version 20.0. Logistic regressions were conducted to analyze the relationship between the variables (vicarious traumas and the impact of traumatic events on a nurses' mental health problems). A confirmatory factor analysis (CFA) was performed to test the validity of the measures. A CFA tests the factor structure of a set of observed variables and the relationship with their latent construct. Therefore, a CFA was performed for the measures/subscales and with more than two items. A CFA was not performed for the coping strategies because the measure did not present a well-defined factor structure, but rather a list of indicators that the authors divided a posteriori into positive and negative strategies to reduce the complexity of the analyses. The factor structure of the measures was estimated using the Comparative Fit Index (CFI) [20], the Tucker Lewis Index (TLI) [21], and the Standardized Root Mean Square Residual (SRMR) [22]. A good fit was reached when the critical value for the CFI and the TLI were 0.90 or higher, and when the SRMR was 0.08 or lower [23]. Cronbach's Alpha was calculated to analyze the reliability of the scales subjected to the CFA. An interaction analysis via the PROCESS macro of the SPSS program was performed to analyze the role of coping strategies as a potential moderator between vicarious traumas and mental health problems. A moderator is a variable that alters the strength of the relationship between an independent variable and a dependent variable. Specifically, we assumed that the positive and negative coping strategies moderated the relationship between vicarious traumas and mental health disorders.

## 3. Results

A priori power analysis using G*Power [24] indicated that a sample size of 134 participants was required to detect medium-sized associations between the study variables with a 95% power (*α* = 5%). We collected 183 completed questionnaires. A post hoc power analysis confirmed that our sample size was sufficient to detect associations between the variables with a power of over 99%.

### 3.1. Demographics

Among the 183 nurses, 77.6% (*n* = 142) were female. Regarding their professional tenure, 36.6% (*n* = 67) of the sample worked as a nurse for less than 5 years, 25.7% (*n* = 47) worked from 5 to 10 years, 15.8% (*n* = 29) from 11 to 20 years, and 21.9% (*n* = 40) worked for more than 20 years. Regarding their working areas, 21.9% (*n* = 40) of nurses worked in a critical area, 36.6% (*n* = 67) in a surgical area, 21.3% (*n* = 39) in a medical area, and 20.2% (*n* = 37) of nurses worked in Services. Regarding their working context, the majority of the sample (59%, *n* = 108) worked in a hospital and 41% (*n* = 75) worked in the community. We considered the sample as a whole since there wasn't a significant difference between the groups (nurses from a hospital or the community) in terms of the variables studied (*p* > 0.05).

### 3.2. Validity of the measures

The results from the CFA showed a good internal validity of the measures. Regarding the impact event scale, the three-factor structure showed a good fit to the data: *χ^2^* (*df* = 189) = 436, CFI = 0.91, TLI = 0.90, and SRMR = 0.05. For the vicarious trauma scale, the model fit the data well: *χ^2^* (*df* = 19) = 45.2, CFI = 0.96, TLI = 0.94, and SRMR = 0.04. Finally, regarding mental health disorders, a three-factor structure showed a good fit to the data: *χ^2^* (*df* = 235) = 512, CFI = 0.91, TLI = 0.90, and SRMR = 0.06.

### 3.3. Descriptive characteristics of the sample

Table 1 shows the means and standard deviations of the study variables and the Cronbach's Alpha of the scales. The results show that the sample reported moderate impact levels for all the three sub-dimensions (Intrusiveness M = 1.73, Hyperarousal M = 1.59, and Avoidance M = 1.47), and high levels of vicarious traumas (M = 4.96). The Cronbach's Alpha values were all above the acceptable level (*α* > 0.70).

**Table 1.** Descriptive characteristics of the sample.

Variables	Mean	Std. Deviation	Likert scale	Cronbach Alpha
IES Intrusiveness	1.73	0.93	0–4 (not at all; extremely)	0.91
IES Hyperarousal	1.59	0.96		0.89
IES Avoidance	1.47	0.82		0.85
Vicarious trauma	4.96	1.22	1–7 (Totally disagree; Totally agree)	0.87
Mental health disorders	1.28	0.88	0–4 (not at all; extremely)	0.96
Positive coping strategies (physical activity, music, reading, yoga)	2.47	0.95	1–4 (never; always)	-
Negative coping strategies (alcohol use, drugs...)	1.35	0.67		-

### 3.4. Regression results

Increased mental health problems (anxiety, somatization, and sleep disorders) were associated with two dimensions of traumatic impact events: intrusiveness and avoidance. Specifically, high levels of intrusive thoughts and avoidance exposed nurses to a 4-fold higher risk of developing mental disorders (W = 4.61, *OR* = 3.96, 95% *CI* 1.13–13.93, *p* = 0.03; W = 10.05, *OR* = 4.44, 95% *CI* 1.77–11.18, *p* < 0.01, respectively). No relationship was found with vicarious traumas (*p* > 0.05) (Table 2).

**Table 2.** Logistic regression results for the relationship between the study variables and mental health disorders.

Dependent variable: Mental health disorders	Wald	*df*	*p*	*B*	Exp (*B*)	95% *CI* for EXP(B)	
						Lower	Upper
IES Intrusiveness	4.61	1	0.03	1.38	3.96	1.13	13.93
IES Hyperarousal	2.89	1	0.09	1.10	3.01	0.84	10.77
IES Avoidance	10.05	1	0.00	1.49	4.44	1.77	11.18
Vicarious trauma	0.07	1	0.80	−0.13	0.88	0.32	2.37
Constant	57.7	1	0.00	−2.55	0.08		

### 3.5. Moderating effects

**Figure 1.**
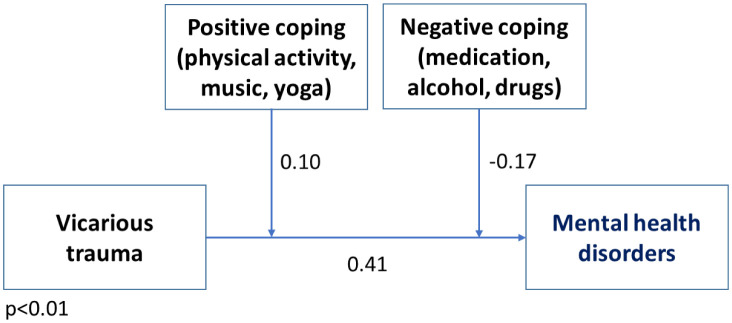
Moderating effect of coping strategies on the relationship between vicarious trauma and mental health problems.

A moderation analysis showed that there was a moderating effect of both positive and negative coping strategies on the relationship between vicarious traumas and the nurses' mental health problems (moderating role of positive coping strategies: coefficient = 0.10, *t* = 2.06, 95% *CI* 0.00–0.20, *p* = 0.041. The model statistics are as follows: *R^2^* = 0.29; *F* = 24.77; *df* = 3179; and *p* < 0.001. The moderating role of the negative coping strategies is as follows: coefficient = −0.17, *t* = −3.47, 95% *CI* −0.27–−0.07, *p* = 0.001. The model statistics are as follows: *R^2^* = 0.44; *F* = 47.09; *df* = 3179; *p* < 0.001) (Figure 1).

**Figure 2.**
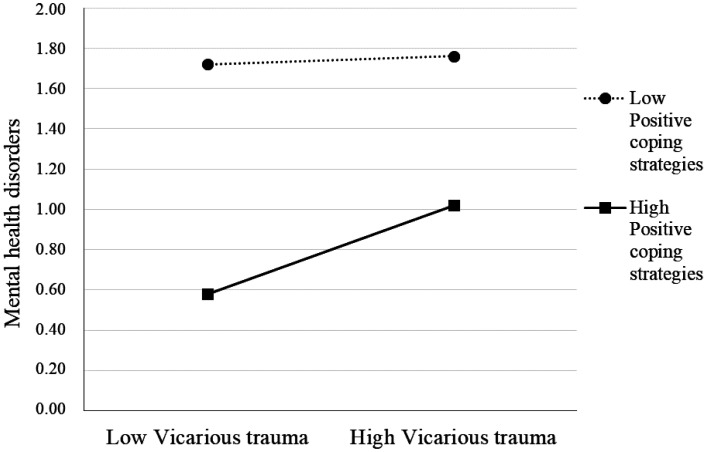
Relationship between vicarious trauma and mental health for low and high levels of moderator (positive coping strategies).

To examine the nature of the interactions, the regression lines for the relationship between vicarious traumas and mental health problems were plotted at high (+1*SD* above the mean) and low (-1*SD* below the mean) levels of coping strategies. Specifically, Figure 2 illustrates that high levels of vicarious traumas increased the nurses' mental health disorders; however, this relationship was weaker when the positive coping strategies (physical activity, reading/music, yoga) were high.

**Figure 3.**
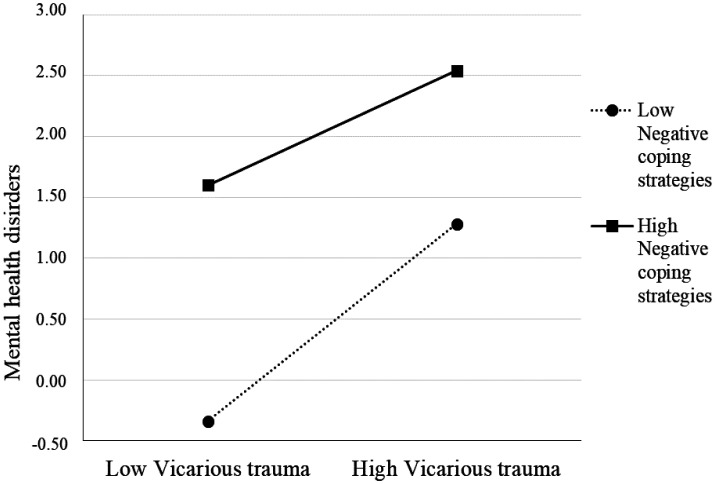
Relationship between vicarious trauma and mental health for low and high levels of moderator (negative coping strategies).

On the contrary, Figure 3 shows that high levels of vicarious traumas increased the nurses' mental health disorders, and this relationship was stronger when the negative coping strategies (medication use, alcohol and drug abuse) were high.

## 4. Discussion

The results showed that vicarious traumas and the impact traumatic events were present among nurses during the pandemic, which affected the workers' wellbeing. Specifically, increased anxiety, somatization, and sleep disorders were associated with high intrusive thoughts and avoidance. Nurses resorted more to using positive coping strategies, such as exercising, listening to music, reading, etc., while rarely resorted to negative coping strategies, such as using prescription and non-prescription drugs and consuming alcohol. The use of negative coping behaviors could provide short-term benefits; however, it can deteriorate mental health in the long run [25]. This finding is consistent with the study of Nie et al. [26], in which nurses mainly used effective support measures to reduce stress such as social support and recreational activities.

Positive coping strategies, such as physical activity, reading/music, and yoga/meditation, were shown to be protective in reducing the effect of vicarious traumas on the nurses' mental health problems. Conversely, negative coping strategies strengthened that relationship and may compromise their quality of working life. In this sense, the organizations should promptly implement measures to enhance the nurses' protection and to lessen the risk of depressive symptoms. In addition, relaxing opportunities at work and individual support based on the workers' specific needs should be planned. These findings provide further support to consider positive coping strategies as an important resource to alleviate psychological distress, thus helping the professional to reduce the negative effects of stress.

Even today, the literature points to the long-term impact of the pandemic on the health of the professionals and their job performance [27],[28]. Moreover, nurses are the main category directly exposed to public health emergencies; maintaining their psychological well-being is essential to cope with the aftermath of the pandemic and in case of future epidemics. Organizational strategies may include increasing briefing and debriefing sessions among the staff to reflect and discuss the negative experiences faced during the working day. Finally, it would be important to provide psychosocial support services for nurses and ongoing efforts to screen for psychological distress. In Italy, to protect the psychological well-being of the professionals, programs have been implemented to help the health workers analyze and communicate their emotional states. Many health organizations have activated specialized mental health clinics to support the professionals in emergencies by providing telephone or Skype support and promoting communication between the professionals and citizens through the web [29].

### 4.1. Limitations and future directions

This study has some limitations that need to be discussed. First, the sample was small and not representative of the nurses who worked in the entire region studied. In this sense, the generalizability should be performed with caution. However, the power analysis showed that our sample size was adequate for our study; therefore, the results are reliable.

Second, due to the restrictions of COVID-19 to limit the contagion, we chose an online administration modality through social groups. This method did not allow us to control the data collection process and may have introduced a sampling bias. In addition, this study lacked a longitudinal design. We conducted a cross-sectional study, which does not allow for causal relationships between variables.

Finally, we used a self-administered questionnaire, which has limitations in terms of a social desirability bias. However, we analyzed the perceptual variables about the nurses' work experiences. For this proposal, self-report questionnaires were appropriate instruments to collect this type of data. As this study included data from the first wave of the pandemic, intervention studies would be welcome at this time to analyze the effect of organizational or individual strategies to increase nurses' well-being.

## 5. Conclusions

This study adds value to the literature by showing an association between intrusive thoughts and avoidance behaviors and mental health problems such as an increased anxiety, somatization, and sleep disturbance. In addition, the study highlighted the role of positive coping strategies as a protective factor by buffering the effects of vicarious traumas on a nurses' mental health. This finding underscores the need for health care organizations to implement interventions that actively promote positive coping skills among the nursing staff. In Italy, several advances have been made to protect the mental health of the professionals during the pandemic, and these advances must be systematically maintained for future risk prevention.

## Use of AI tools declaration

The authors declare they have not used Artificial Intelligence (AI) tools in the creation of this article.
